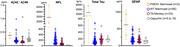# Characterizing plasma biomarkers of Alzheimer’s disease in three non‐human primate species: marmosets, titi monkeys, and capuchins

**DOI:** 10.1002/alz.093030

**Published:** 2025-01-09

**Authors:** Emily S. Rothwell, Allison Lau, Meghan Sosnowski, Karen L. Bales, Sarah Brosnan, Afonso C Silva, Stacey J Sukoff Rizzo

**Affiliations:** ^1^ University of Pittsburgh School of Medicine, Pittsburgh, PA USA; ^2^ California National Primate Research Center, Davis, CA USA; ^3^ University of California Davis, Davis, CA USA; ^4^ Georgia State University, Atlanta, GA USA; ^5^ University of Pittsburgh, Pittsburgh, PA USA

## Abstract

**Background:**

Studies of aging in non‐human primates are important to elucidate primate‐specific mechanisms underlying human aging, including pathological trajectories like Alzheimer’s disease (AD). Evidence of AD‐like brain aging has been reported across the primate order including amyloid beta (AB) deposits, but blood‐based biomarkers are less well‐studied. The goal of this project was to explore the use of validated assays for plasma biomarkers in two new non‐human primate species: coppery titi monkeys (Plecturocebus cupreus) and brown capuchins (Sapajus apella). We aimed to 1) assess the utility of the assay, 2) establish normative values, and 3) explore age‐related differences in biomarker levels. For each species, we assessed biomarkers sensitive to physiologic aging and pathological conditions including neurodegeneration/neuronal injury (neurofilament light chain, NfL), neuroinflammation (glial fibrillary acidic protein, GFAP), neuropathology (AB40, AB42, pathological tau).

**Method:**

Plasma samples were obtained from males and females of adult common marmosets (Callithrix jacchus) (n=85), titi monkeys (n= 33), and capuchins (n=19), and evaluated using MesoScale Discovery (MSD): AB Peptide 4G8 Panel & Neurology Panel 1multi‐plex ELISAs. Samples from genetically engineered marmosets with early onset familial mutations in the presenilin 1 (PSEN1) gene were included as positive controls and benchmarks (Sukoff Rizzo et. al, 2023).

**Result:**

AB40, AB42, Tau, GFAP, and NfL were detectable in plasma from titi monkeys and capuchins. Biomarker levels across ages of each species had comparative values and overlapping ranges with wildtype (WT) adult marmosets. Consistent with previous data PSEN1 knock‐in marmosets had the expected increase in plasma AB42 relative to WT, with some individuals of each species showing naturally elevated levels. For both marmosets and titis, NfL was found to increase with advancing age. Titi monkeys also showed increases in AB40 and GFAP with age.

**Conclusion:**

The present data are the first to demonstrate normative values for plasma biomarkers of neurodegeneration, neuroinflammation, and neuropathology in capuchin and titi monkeys. Preliminary results show comparative values and aging trajectories across three species of non‐human primates. Ongoing studies are evaluating additional individuals and species, to longitudinally track aging which will facilitate understanding primate‐specific mechanisms underlying healthy human aging as well as AD.